# Defective DNAM-1 Dependent Cytotoxicity in Hepatocellular Carcinoma-Infiltrating NK Cells

**DOI:** 10.3390/cancers14164060

**Published:** 2022-08-22

**Authors:** Stefania Mantovani, Stefania Varchetta, Dalila Mele, Roberta Maiello, Matteo Donadon, Cristiana Soldani, Barbara Franceschini, Guido Torzilli, Giuseppe Tartaglia, Marcello Maestri, Gaetano Piccolo, Matteo Barabino, Enrico Opocher, Stefano Bernuzzi, Mario U. Mondelli, Barbara Oliviero

**Affiliations:** 1 Division of Clinical Immunology-Infectious Diseases, Department of Medicine, Fondazione IRCCS Policlinico San Matteo, 27100 Pavia, Italy; 2 Department of Molecular Medicine, University of Pavia, 27100 Pavia, Italy; 3 Department of Biomedical Science, Humanitas University, Pieve Emanuele, 20090 Milan, Italy; 4 Department of Hepatobiliary and General Surgery, IRCCS Humanitas Research Hospital, Rozzano, 20089 Milan, Italy; 5 Laboratory of Hepatobiliary Immunopathology, IRCCS Humanitas Research Hospital, Rozzano, 20089 Milan, Italy; 6 Division of General Surgery 1, Department of Surgery, Fondazione IRCCS Policlinico San Matteo, 27100 Pavia, Italy; 7 Unit of HepatoBilioPancreatic and Digestive Surgery, Department of Health Sciences, San Paolo Hospital, University of Milan, 20142 Milan, Italy; 8 Immunohematology and Transfusion Service, Department of Diagnostic Medicine, Fondazione IRCCS Policlinico San Matteo, 27100 Pavia, Italy; 9 Department of Internal Medicine and Therapeutics, University of Pavia, 27100 Pavia, Italy

**Keywords:** natural killer cells, DNAM-1, PVR, hepatocellular carcinoma

## Abstract

**Simple Summary:**

Hepatocellular carcinoma (HCC) is the most common form of primary liver cancer and the fourth leading cause of cancer-related deaths worldwide. Although therapeutic options have improved in the last few years, mortality remains disturbingly high. The key role of innate immunity, particularly of natural killer (NK) cells, in tumor surveillance and response is well established. The anti-tumor NK cell activity is modulated by interactions between NK cells activating or inhibiting receptors and their ligands, expressed or released by tumor cells. Alterations in these networks lead to inadequate NK cell responses and a lack of cancer control. In our study, we focus on NK cells activating receptor DNAM-1 and its ligand CD155, expressed in HCC cells. We provide evidence of impaired NK cytotoxic function as a result of altered receptor/ligand axis. We conclude that this may represent a tumor escape mechanism and a possible target for new immunotherapeutic approaches to HCC treatment.

**Abstract:**

Background: Natural killer (NK) cells play a key role in immune surveillance and response to tumors, their function regulated by NK cell receptors and their ligands. The DNAM-1 activating receptor recognizes the CD155 molecule expressed in several tumor cells, such as hepatocellular carcinoma (HCC). This study aims to investigate the role of the DNAM-1/CD155 axis in mediating the NK cell response in patients with HCC. Methods: Soluble CD155 was measured by ELISA. CD155 expression was sought in HCC cells by immunohistochemistry, qPCR, and flow cytometry. DNAM-1 modulation in NK cells was evaluated in transwell experiments and by a siRNA-mediated knockdown. NK cell functions were examined by direct DNAM-1 triggering. Results: sCD155 was increased in sera from HCC patients and correlated with the parameters of an advanced disease. The expression of CD155 in HCC showed a positive trend toward better overall survival. DNAM-1 downmodulation was induced by CD155-expressing HCC cells, in agreement with lower DNAM-1 expressions in tumor-infiltrating NK (NK-TIL) cells. DNAM-1-mediated cytotoxicity was defective both in circulating NK cells and in NK-TIL of HCC patients. Conclusions: We provide evidence of alterations in the DNAM-1/CD155 axis in HCC, suggesting a possible mechanism of tumor resistance to innate immune surveillance.

## 1. Introduction

Poliovirus receptor (PVR, CD155) is considered a member of both the immunoglobulin superfamily, presenting an immunoglobulin domain V, a C1-like domain, and a C2 domain in the extracellular region [1] and of the nectin-like family, as it shares with this family three conserved motifs in the IgV domain [2]. CD155 is a multi-functional protein. Although it was first described as a poliovirus entry receptor [3] for its ability to allow attachment and entry of poliovirus in susceptible cells, PVR is also involved in several physiological functions, as a mediator of cellular adhesion, contact inhibition, cellular motility, proliferation, and survival [4]. The mRNA derived from the CD155 gene transcription is alternatively spliced into four isoforms: α and δ are transmembrane proteins, whereas β and γ are secreted as soluble forms, lacking the intracellular domain [5]. Unlike the CD155δ, which has a shorter C-terminus intracellular domain, the PVRα form contains an immunoreceptor tyrosine-based inhibitory motif (ITIM), responsible for signal transduction [6]. The soluble forms consist of the extracellular domain of the CD155 molecule and contribute to neutralizing the binding and entry of poliovirus into the host cells in vitro [7]. Although PVR expression is low or absent in most healthy tissues, in several human cancers, an upregulation of both membrane and soluble forms was observed and correlated with poor prognosis. For instance, in colorectal cancer, immunohistochemical (IHC) and mRNA analysis revealed an increased level of the CD155 protein compared, with tumor-free colon mucosa [8]. IHC analysis also showed a powerful expression of PVR in primary lung adenocarcinoma tissue [9]. Upregulated PVR mRNA and protein expression were found in primary and metastatic melanomas and correlated with disease progression [10]. The limited CD155 expression was found in non-neoplastic cells from several human pancreatic cancers, while it was abundantly expressed in cancer cells [11]. Moreover, patients with high PVR expression (over 50% CD155 positivity) showed poorer postoperative prognosis than those with low expression [11]. Again, when the CD155 isoforms were analyzed by quantitative real-time PCR (qPCR) in several tumor tissues, such as colorectal, gastric, and breast cancers, the expression of CD155α and CD155β was higher than that in the adjacent non-tumor tissues [12]. Levels of soluble CD155 (sCD155) were significantly higher in the sera of 262 patients with various cancers, including lung, esophageal, gastric, colorectal, bile-duct, pancreatic, breast, ovarian, endometrial, and cervical cancers compared with healthy donors, suggesting that sCD155 could be a potential pan-tumor biomarker [12]. Interestingly, the serum of patients with HCC also displayed a higher level of sCD155 compared to patients with intrahepatic cholangiocarcinoma or with liver diseases, such as cirrhosis or hepatitis B virus infection [13]. Among its several functions, CD155 regulates immune responses, which could play a critical role in immune surveillance of tumors. Indeed, PVR is the common ligand for CD96, T cell immunoreceptor with IG and ITIM domains (TIGIT), and DNAX-associated molecule-1 (DNAM-1) receptors. CD96 and TIGIT deliver inhibitory signals, whereas DNAM-1 delivers activating signals [14].

DNAM-1 is found in CD8+ T cells, cytokine induced killer (CIK) cells, and NK cells, [15] where it induces the release of cytokines, such as interferon (IFN)-γ, and stimulates their cytotoxic activity that ultimately lead to the apoptosis of tumor targets or infected cells [15,16].

The importance of the DNAM-1/CD155 axis in tumor control was first described in a mouse lymphoma model where a high expression of CD155 induced a robust response mediated by NK cells in a DNAM-1 dependent mechanism (reviewed in [16]). Moreover, mice lacking DNAM-1 were more susceptible to fibrosarcoma formation [17] and to metastasis spread due to defective NK cytotoxicity [18]. In humans, freshly isolated neuroblast susceptibility to lysis directly correlates with the surface expression of CD155 and either DNAM-1 or PVR masking with monoclonal antibodies, resulted in strong inhibition of tumor cell lysis [19]. A key role for DNAM-1 receptor engagement by CD155 was also demonstrated in the control of myeloma development [20].

Hepatocellular carcinoma (HCC) accounts for approximately 90% of primary liver cancers, usually developing in the background of cirrhosis. Besides surgery, liver transplantation, or loco-regional approaches, options for HCC treatment include first line (VEGF inhibitors) and second line (immune check-point inhibitors, ICI) medications, but mortality remains high, with an average 5-year survival rate of 20% [21].

Several studies pointed to the importance of innate immunity in the control of cancer, including HCC. In particular, changes in NK cell frequency and phenotype were described during HCC development in a transgenic mouse model of aggressive human liver cancer [22], and evidence showed a positive correlation between the frequency of circulating and intrahepatic NK cells and survival in patients with HCC [23]. Several alterations in phenotype and function of circulating and intratumoral NK cells are observed, being the NKp30- and NKG2D-mediated functionality deficient [24,25]. Interestingly, altered expression of CD155 in HCC tissues correlates with the prognosis of the disease. In fact, patients with HCC expressing CD155 have better overall survival after surgery than those with negative CD155 expression [26,27]. Thus, the evidence supporting a potential role of CD155 expression and the NK cell function in the HCC control prompted us to investigate the CD155/DNAM-1 axis in this disease.

## 2. Materials and Methods

### 2.1. Patients and Biological Materials

Paired peripheral blood and resected HCC specimens, along with matched non-tumor (NT) surrounding tissue, were obtained from patients admitted to Fondazione IRCCS Policlinico San Matteo, Pavia, IRCCS Humanitas Research Hospital, and ASST Santi Paolo e Carlo Hospital, Milan, Italy. Peripheral blood from a cohort of patients with cirrhosis but without HCC was obtained from Fondazione IRCCS Policlinico San Matteo. The main patient characteristics are listed in supplementary Table S1. Healthy donors (HD) comprised 7 females (23%) and 23 males (77%), with a median age of 50 years (range 36–71 years). Written informed consent was obtained from each individual. The study protocol complies with the ethical guidelines of the 1975 Declaration of Helsinki and was approved by our institutional ethical committee (protocol numbers: 201430031379, 20190081830).

### 2.2. ELISA

Serum levels of sCD155 were measured by a Human Poliovirus Receptor (PVR) ELISA kit (CUSABIO, cat. Number: CSB-EL019093HU) in patients and HD, according to the manufacturer’s instructions.

### 2.3. Immunohistochemistry

CD155 expression was evaluated by immunohistochemistry in 2 mm paraffin-embedded tissue sections. After deparaffinization and rehydration of the sections, antigen demasking was performed in an EDTA buffer (pH 9.0) at 98 °C (Dako). Endogenous peroxidase activity and unspecific binding sites were blocked with peroxidase-blocking solution for 15 min at room temperature (Dako). Slides were washed and incubated with an anti-CD155 antibody (clone 300907, 5 ug/mL, R&D) overnight. An Envision peroxidase (HRP)-conjugated secondary antibody (Dako) was applied for 30 min, and the positivity was developed with 3,3′-diaminobenzidine (Dako). Tissue sections were counterstained with Mayer’s haematoxylin. As a negative control, the primary antibody was omitted. Images were captured by light microscopy using an Olympus BX51 microscope.

### 2.4. DNAM-1 Downregulation Experiments

Freshly peripheral blood mononuclear cells (PBMCs) (1 × 10^5^) from HDs were co-cultured overnight alone, in the presence of 1 × 10^5^ CD155+ Huh7.5 cell line or primary HCC cells, and with supernatants of Huh 7.5 or HCC primary cell cultures. The co-cultures were also performed in the Transwell^®^ system (Corning, NY, USA). The percentage of DNAM-1+ NK cells and the expression of DNAM-1 in NK cells were evaluated in each condition by flow cytometry. HD NK cells were enriched freshly from PBMCs by using the EasySep^TM^ Human NK cell Enrichment Kit (Stemcell Technologies, Vancouver, BC, Canada), according to the manufacturer’s instructions.

### 2.5. siRNA-Mediated Knockdown

Silencer^®^ Select siRNA (Thermo Fisher Scientific, Waltham, MA, USA), designed to knock down the CD155 gene expression, and a Silencer^®^ Select negative control siRNA were used to a final RNA concentration of 10 nM. Sequences are: (5′->3′) sense GGCUAUAAUUGGAGCACGAtt and antisense UCGUGCUCCAAUUAUAGCCtg.

The HepG2 cell line and HCC primary cells were reverse transfected following the manufacturer’s instructions. siRNA knockdown was assessed by flow cytometry. PBMCs (1 × 10^5^) from HDs were co-cultured overnight alone, with 1 × 10^5^ siRNA-CD155 transfected HepG2 or HCC primary cells, and with siRNA-control transfected HepG2 or HCC primary cells. The DNAM-1 modulation was evaluated as a percentage of DNAM-1+ NK cells and mean fluorescence intensity of DNAM-1 in NK cells in each condition by flow cytometry.

### 2.6. Isolation of Peripheral Blood Mononuclear Cells and Tissue-Infiltrating Lymphocytes

PBMCs and tissue-infiltrating lymphocytes were isolated as previously described [24]. Briefly, PBMCs were obtained by Lympholyte (Cedarlane, Burlington, ON, Canada) density gradient (1.0770) centrifugation following the manufacturer’s instructions. To isolate liver-infiltrating lymphocytes (LIL) and tumor-infiltrating lymphocytes (TIL), tissue samples were treated by enzymatic and mechanical dissociation with the human Tumor Dissociation Kit and gentle MACS Dissociator (Miltenyi Biotec, Bergisch Gladbach, Germany), according to the manufacturer’s instructions. The cell suspension was filtered in a 70 µm cell strainer and centrifuged twice at 50× *g* for 2 min to obtain LIL and TIL cells. The supernatant containing lymphocytes was washed and cryopreserved in liquid N2.

### 2.7. Primary HCC Cell Cultures

HCC cells were obtained from enzymatic and mechanical dissociation of tissue samples by using the human Tumor Dissociation Kit and gentle MACS Dissociator (Miltenyi Biotec). The cells obtained were subsequently plated in tissue culture flasks (Corning) with Dulbecco’s Modified Eagle Medium (Thermo Fisher Scientific), supplemented with 10% fetal bovine serum (FBS, HyClone, GE Healthcare, South Logan, UT, USA), 1% antibiotic antimycotic solution (100 U/mL penicillin, 0.1 µg/mL streptomycin, 0.25 µg/mL amphotericin B) (Sigma-Aldrich, St. Louis, MO, USA), and 1% non-essential amino acids (Thermo Fisher Scientific). The surface expression of CD155 was examined in patient-derived tumor cells and in the Huh 7.5 cell line by labeling cells with the anti-CD155 PE (Miltenyi Biotec) monoclonal antibody (mAb).

### 2.8. Phenotypic and Functional Analysis

The phenotype and function of NK cells from PBMCs, and LIL and TIL cells were analyzed using a 12-colour FACSCelesta (BD Biosciences, San Diego, CA, USA) flow cytometer. The NK cells were identified as CD3neg and CD56pos (anti-CD3 BV510 and anti-CD56 BB700 mAbs, BD Biosciences). DNAM-1 expression was measured by labeling cells with the anti-DNAM-1 BB515 (BD Biosciences). The gating strategy to identify DNAM-1+ cells among NK cells, NK-LIL, and NK-TIL is showed in supplementary Figure S1. In the functional assays, PBMCs, and LIL and TIL cells were unstimulated or stimulated overnight with IL-15 (20 ng/mL for 15 h: Peprotech, London, UK). After incubation, cells were harvested and co-cultured with matched primary HCC or K562 target cells in a 1:1 effector: target cell ratio for 4 h at 37 °C in the presence of anti-CD107a and a BD GolgiPlug™ Protein Transport Inhibitor (Becton Dickinson). To assess the cytokine production, cells were subsequently fixed with BD Cytofix/Cytoperm (BD Biosciences) and permeabilized with the BD Perm/Wash buffer (BD Biosciences) in the presence of anti-IFN-γ BV421 (BD Biosciences) and, according to the manufacturer’s instructions, cells were washed, fixed in CellFix solution (BD Biosciences), and analyzed. In the reverse antibody-dependent cellular cytotoxicity (rADCC) assay, unstimulated or IL-15 stimulated PBMCs, and TIL and LIL cells, were incubated with FcγR+ P815 murine target cells with a 1:1 effector:target ratio, in the presence of the anti-DNAM-1 unconjugated mAb (BioLegend, San Diego, CA, USA). Degranulation and IFN-γ production were evaluated as described above. Data analysis was performed using the Kaluza 2.1 software (Beckman Coulter, Brea, CA, USA).

### 2.9. RNA Extraction and qPCR

The tissue RNA extraction was performed using a TRIzol reagent (Thermo Fisher Scientific) with a gentleMACS Dissociator (Miltenyi Biotec), and with an RNeasy Plus Mini kit and DNAse treatment on a column (Qiagen, Hilden, Germany), according to the manufacturer’s instructions. First-strand cDNA was synthesized from 2 μg of total RNA using a High-Capacity cDNA Reverse Transcription Kit, following the manufacturer’s instructions (Thermo Fisher Scientific). The SsoAdvanced Universal SYBR Green Supermix (BioRad, Hercules, CA, USA) was used, and qPCR data were analyzed using the 2−ΔCt method. CD155 mRNA expression was normalized to the ß2-microglobulin (ß2M) gene. The following primers were used: ß2M forward 5′-ACACTGAATTCACCCCCACT-3′ and reverse 5′-GCTTACATGTCTCGATCCCACTT -3′; CD155-α forward 5′-TGTTCCCGTGAGGTCCTTTG-3′, reverse 5′-ACATGCCCATTAGCTGAGGC-3′; CD155-β forward 5′-CCGTCCTGTGGACAAACCAA-3′, reverse 5′-GGCATGCTCTGTACCTGAGT-3′; CD155-δ forward 5′-TGTTCCCGTGAGGTCCTTTG-3′, reverse 5′-AATTACGGCAGCTCTGGTGA-3′; CD155-γ 5′-CCGTCCTGTGGACAAACCA-3′, reverse 5′-TCTGTACCTTTGACCTGGACG-3′; CD155 5′-AATGCCTCGCTGAGGATGTT-3′, reverse 5′-CTCCAGTGAGCTGGACCTTC-3′ (Primm, Milan, Italy).

### 2.10. Statistical Analysis

Statistical analysis was performed using the GraphPad Prism 6 software (GraphPad, La Jolla, CA, USA). Data distributions among groups were checked for normality (D’Agostino–Pearson test) before any analysis. Since the distribution was consistently not normal, we used non-parametric tests for all our analyses: the Wilcoxon matched-pairs signed-rank test for paired data, and the Mann–Whitney U test for unpaired data to compare two groups; and the non-parametric Kruskal–Wallis or Friedman tests to compare more than two groups of non-matched or matched data, respectively. Data correlations were assessed using Spearman’s correlation test. Kaplan–Meier curves were used to analyze differences in survival, and were compared using the log-rank test. For this purpose, overall survival was chosen because it was considered as the most reliable and unbiased endpoint in HCC patients. A *p* value ≤ 0.05 was deemed statistically significant.

## 3. Results

### 3.1. Patients with Advanced HCC Have Higher Concentrations of Serum CD155

**Figure 1:**
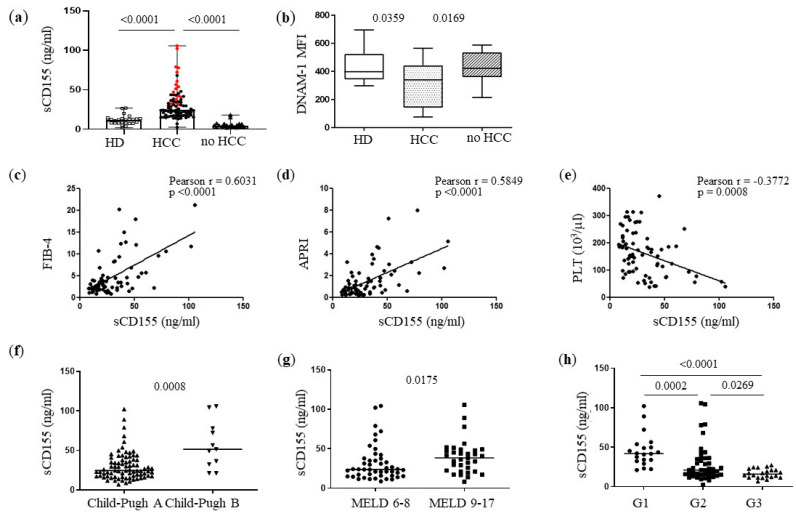
Soluble CD155 correlates with advanced disease in HCC patients. (**a**) Soluble CD155 (sCD155) was tested in sera of 30 HD, 88 HCC and 22 no HCC subjects. HCC patients with an advanced disease are indicated as red dots. (**b**) DNAM-1 expression (mean fluorescence intensity, MFI) in NK cells of 16 HDs, 30 HCC subjects, and 16 no HCC subjects. (**c**–**e**) Correlation between sCD155 concentration in serum form HCC patients and FIB-4, APRI and number of platelets (PLT). (**f**,**g**) sCD155 in HCC stratified according to Child–Pugh (A, n = 75; B n = 11) and MELD (6–8, n = 46; 9–17, n = 34) disease indicators. (**h**) sCD155 in HCC stratified according to the grade of tumor differentiation: G1, G2, and G3 (well, moderately and poorly differentiated, respectively); G1: n = 21; G2, n = 43; G3, n = 22. Middle bars represent median values, box plots are 25% and 75% percentiles, whiskers are minimum and maximum values. The non-parametric Kruskal–Wallis or Mann–Whitney U tests were used to compare data in (**a**,**b**) and (**f**–**h**) panels. The non-parametric Spearman’s test was used to correlate sCD155 and clinical parameters ((**c**–**e**) panels).

Soluble CD155 was shown to be increased in several solid tumor settings [12], including HCC [13]. We therefore measured serum CD155 levels in patients with cirrhosis and HCC, patients with cirrhosis and no cancer (no HCC), and healthy donors (HDs). Serum levels of sCD155 were significantly higher in patients with HCC compared with both HDs and no HCC subjects (Figure 1a) and this was accompanied by a simultaneously lower expression of DNAM-1 in NK cells from HCC patients (Figure 1b). Interestingly, sCD155 concentrations were higher in sera from HCC patients with an advanced disease. Indeed, sCD155 directly correlated with non-invasive markers of fibrosis, such as the fibrosis-4 (FIB-4) score (Figure 1c) and the aspartate aminotransferase to platelet ratio index (APRI) (Figure 1d). As expected, platelet count was instead inversely correlated with sCD155 (Figure 1e). HCC patients with advanced cirrhosis, i.e., those with Child–Pugh B (Figure 1f) and model of end-stage liver disease (MELD) scores between 9 and 17 (Figure 1g) showed higher sCD155 concentrations. Of note, when HCC patients were stratified according to the grade of tumor differentiation, there was a tumor grade-dependent sCD155 decrement in sera from patients with moderately (G2) and poorly differentiated (G3) tumors compared with well-differentiated (G1) tumors (Figure 1h). When a median value of sCD155 (26.83 ng/mL) was identified as the cut-off to discriminate between patients with high or low sCD155 levels, we found that the former mainly stratified in the G1 group, whereas the latter mainly stratified in the G3 group (Figure S2). There was no correlation between serum levels of sCD155 in any HCC patients and clinical scores.

### 3.2. Hepatocellular Carcinoma Tissue Expresses Variable Levels of CD155

**Figure 2:**
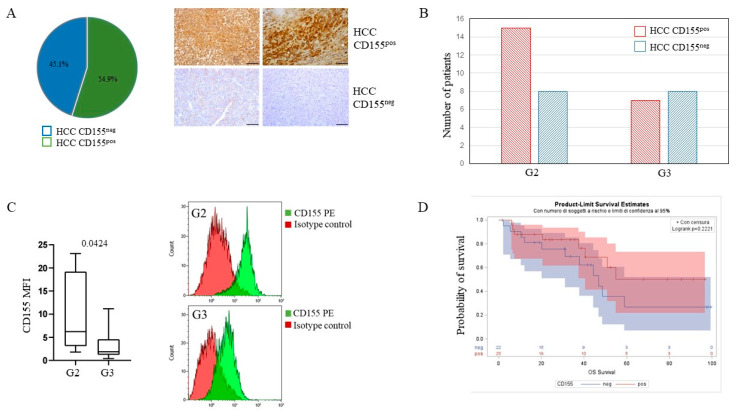
CD155 expression on tumor tissues. (**A**) Distribution of CD155^pos^ and CD155^neg^ HCC specimens and representative images of immunohistochemical analysis (n = 51). Representative images of CD155 positive (upper row) and negative (lower row) expressions in HCC tumor tissues at 10X magnification, bars: 100 μm. (**B**) Distribution of the same CD155^pos^ and CD155^neg^ HCC patients stratified according to tumor grading (moderate and poorly differentiated, G2 and G3 respectively). (**C**) Expression of CD155 in HCC primary cells from G2 (n = 7) and G3 tumors (n = 10) evaluated by flow cytometry (MFI, calculated as CD155 MFI-isotype control MFI). Representative histograms of CD155 expression in HCC primary cells derived from a G2 and a G3 tissue. (**D**) Overall survival of HCC patients according to CD155 expression.

To assess whether higher sCD155 serum concentrations could result from a higher release of CD155 by tumor cells, we sought membrane CD155 expression in HCC tissue in a proportion of patients. Immunohistochemical analysis of the tumor and surrounding liver specimens from HCC patients revealed that, among 51 HCC samples, 28 (55%) were CD155pos and 23 (45%) were CD155neg (Figure 2A). When patients were stratified according to the tumor differentiation stage, we found that CD155 was more frequently expressed in G2 compared with G3 tumors (Figure 2B). Expression of CD155 was also analyzed in HCC cells obtained from resected patients after the enzymatic and mechanical dissociation of tumor tissue and a higher intensity of CD155 expression was found in the G2 compared with the G3 tumors (Figure 2C). Notably, there was a trend in patients carrying a CD155pos tumor toward a higher, though not statistically significant, overall survival (Figure 2D). To understand if the variability of CD155 expression could be due to the prevalence of one of the CD155 isoforms with the consequent preponderance of the soluble or transmembrane form, we measured mRNA levels of the CD155 isoforms in HCC and matched NT tissues. Although there was a trend toward higher mRNA in HCC tissue compared with NT (Figure S3a), transmembrane (trCD155) as well as soluble (sCD155) CD155 isoform mRNA levels were comparable in NT, and matched HCC tissue (Figure S3b). No statistically significant differences in single mRNA isoform levels were observed between HCC and NT, despite there being a trend in HCC toward higher mRNA levels of all isoforms analyzed (Figure S3c–f).

### 3.3. CD155 Directly Down-Modulates DNAM-1 Expression

**Figure 3:**
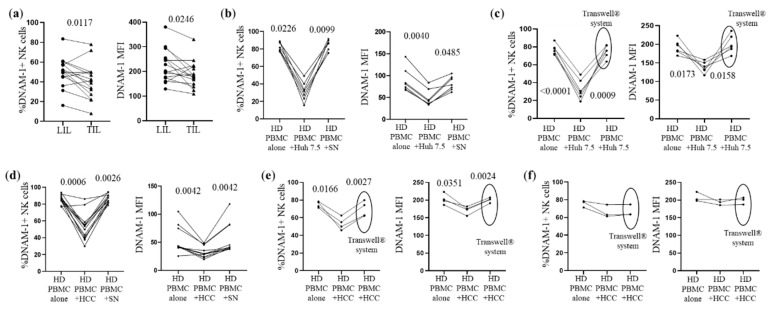
DNAM-1 is downregulated upon exposure to tumor cells. (**a**) Proportion of DNAM-1+ NK cells in LIL and matched TIL and DNAM-1 expression (as MFI) measured ex vivo by flow cytometry. The paired t-test was used to compare data. (**b**) The frequency of DNAM-1+ NK cells and DNAM-1 MFI was analyzed in NK cells from seven HDs in the presence or absence of Huh 7.5 cells or cell-free supernatant (SN) from Huh 7.5 cell cultures. (**c**) The frequency of DNAM-1+ NK cells and DNAM-1 MFI were determined in PBMCs, after exposure to Huh 7.5 cells or in the Transwell^®^ system to prevent contact with Huh 7.5 cells (HD, n = 6). (**d**) Downregulation was assessed after co-culture of PBMCs from 13 HDs with 5 primary HCC cells or with HCC cell culture SN. (**e**) DNAM-1 downmodulation in NK cells from four HDs co-cultured with primary HCC cells expressing CD155. (**f**) The experiments were repeated using primary HCC cells expressing low levels of CD155. The non-parametric Friedman test for matched data was used for comparisons.

When we studied the liver compartment, we observed a lower percentage of DNAM-1+ NK cells in TIL. Moreover, NK-TIL showed a lower DNAM-1 expression that matched NK-LIL (Figure 3a and Supplementary Materials Figure S4a). To assess whether CD155 expressed inn HCC cells could affect the expression of DNAM-1 in NK cells, we performed co-culture experiments with the Huh 7.5 hepatoma cell line, which expresses CD155 (Figure S5a). DNAM-1 significantly decreased in NK cells after exposing PBMCs to the Huh 7.5 cell line (Figure 3b and Supplementary Materials Figure S4b). In contrast, the Huh 7.5 culture supernatant was unable to down-modulate the DNAM-1 expression (Figure 3b and Supplementary Materials Figure S4b), showing that cell–cell contact was indeed required. The requirement for cell–cell contact was confirmed by transwell (Transwell^®^ system) experiments. Indeed, when the contact between PBMCs and Huh 7.5 was prevented by transwell experiments, DNAM-1 expression was restored in NK cells (Figure 3c, Supplementary Materials Figure S4c).

Exposure to primary HCC cell lines expressing CD155 (Figure S5b) determined a similarly significant DNAM-1 downmodulation in NK cells (Figure 3d and Supplementary Materials Figure S6a). Again, in the Transwell experiments, the prevention of HCC cell and PBMC contact induced a restoration of DNAM-1 levels (Figure 3e and Supplementary Materials Figure S6b). We checked that a lower expression of CD155 in HCC cells (Figure S5c) did not induce significant DNAM-1 downmodulation (Figure 3f and Supplementary Materials Figure S6c). To verify if the observed DNAM-1 downmodulation was caused by an indirect effect mediated by other immune cells included in PBMCs, we performed experiments using enriched NK cells (Figure S7a). Identical findings were indeed obtained with purified NK cells, since having both the frequency of DNAM-1+ NK cells (Figure S7b,c) and the NK cell expression of DNAM-1 significantly decreased after co-culture, with CD155-expressing Huh 7.5 cells or primary HCC cells (Figure S7d,e).

**Figure 4:**
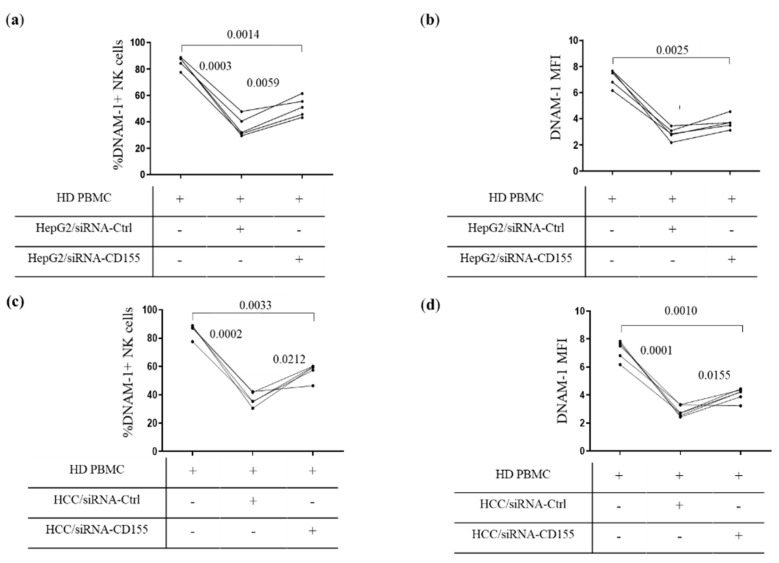
Tumor-siRNA-CD155 transfected cells modulate DNAM-1 expression. (**a**) Frequency of DNAM-1+ NK cells and (**b**) DNAM-1 expression (MFI) in PBMCs from 5 HDs exposed to siRNA-CD155- or siRNA-control-transfected HepG2 cells. (**c**) Frequency of DNAM-1 positive NK cells and (**d**) MFI were evaluated in NK cells of PBMCs from five HDs after exposure to primary HCC cells transfected with siRNA-CD155 or siRNA-control. Matched data were analyzed by a parametric ANOVA test.

CD155-induced DNAM-1 downregulation was also confirmed by siRNA-mediated knockdown. Surface CD155 expression was evaluated in the HepG2 cell line after transfection with CD155 siRNA or the negative control. siRNA-mediated knockdown of CD155 reduced CD155 expression in HepG2 cells (Figure S5d). After exposure of PBMCs from HDs to siRNA-CD155 HepG2 cells, the frequency of DNAM-1+ NK cells (Figure 4a and Supplementary Materials Figure S8a) and the expression of DNAM-1 (Figure 4b and Supplementary Materials Figure S8b) were both significantly increased compared to the siRNA-negative control.

We also evaluated the effect of the siRNA-CD155 transfection in the primary HCC cell line and found that siRNA reduced CD155 expression (Figure S5e). This resulted in an increased frequency of DNAM-1+ NK cells (Figure 4c and Supplementary Materials Figure S8c), as well as a higher DNAM-1 expression (Figure 4d and Supplementary Materials Figure S8d).

### 3.4. Defective DNAM-1-Mediated Cytotoxicity in Peripheral and Tumor-Infiltrating NK Cells of HCC Patients

**Figure 5:**
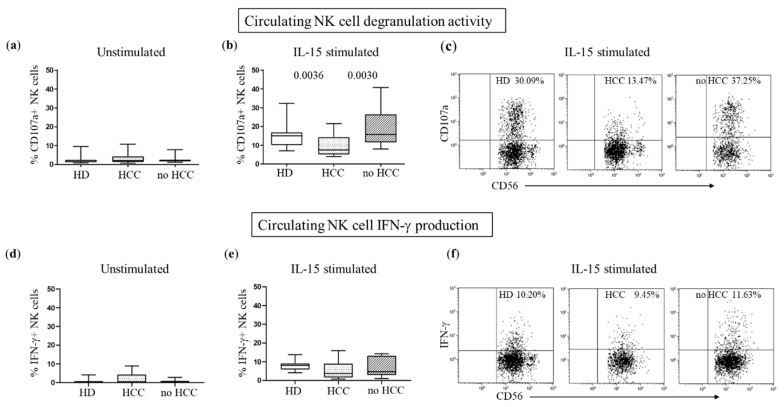
Decreased DNAM-1-mediated cytotoxicity in circulating NK cells. A reverse antibody-dependent cytotoxicity (rADCC) assay was used to test DNAM-1 receptor-mediated NK cell functions. Degranulation activity (%CD107a+ NK cells) of circulating NK cells from 11 HDs, 20 HCC subjects, and 11 no HCC subjects was analyzed before (**a**) and after (**b**) IL-15 stimulation. (**d**,**e**) IFN-γ production was tested in NK cells from 11 HD, 15 HCC subjects, and 11 no HCC subjects, under the same experimental conditions. Middle bars represent median values, box plots are 25% and 75% percentiles, whiskers are minimum and maximum values. The non-parametric Kruskal–Wallis test was used to compare data. Representative dot plots of degranulation (**c**) and IFN-γ production (**f**) of stimulated NK cells from HDs, HCC subjects, and no HCC subjects are also shown.

To assess whether the CD155/DNAM-1 axis was altered in HCC patients, we examined NK cell function by reverse antibody-dependent cellular cytotoxicity (rADCC) assay, in which unstimulated or IL-15-stimulated PBMCs, TIL, and matched LIL cells, were incubated with FcγR+ P815 murine target cells in the presence of an anti-DNAM-1 specific mAb. In this way, we could analyze cytotoxicity and IFN-γ production specifically mediated by DNAM-1. As shown in Supplementary Figure S9, anti-DNAM-1 mAb was able to increase NK cell degranulation compared with IL-15 stimulation alone. Baseline peripheral blood NK cell degranulation and cytokine release were comparable in HDs, HCC patients, and no HCC patients (Figure 5a,d). However, after treatment with IL-15, we observed an increased proportion of CD107a+ NK cells that remained significantly lower in HCC compared with HDs and no HCC patients (Figure 5b,c). Interestingly, no differences were found in IFN-γ production (Figure 5e,f).

**Figure 6:**
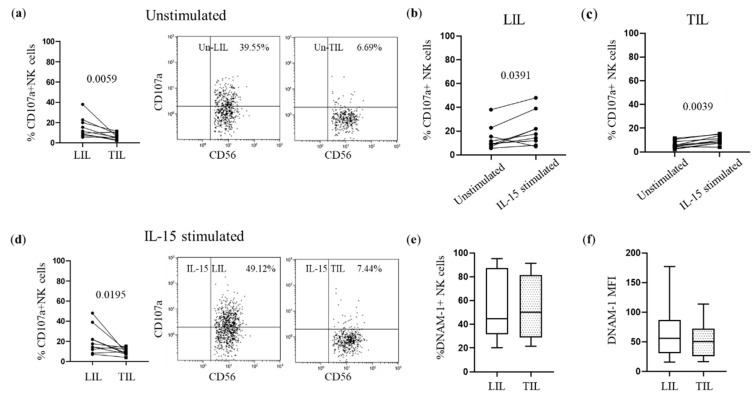
Impaired NK-TIL degranulation activity mediated by DNAM-1. (**a**,**d**) Degranulation of NK-LIL and matched NK-TIL was analyzed after DNAM-1 triggering in rADCC assays in 10 HCC patients before and after IL-15 stimulation. Representative dot plots showing unstimulated (Un-LIL, Un-TIL) or IL-15 stimulated (IL-15 LIL, IL-15 TIL) cells. (**b**,**c**) IL-15 stimulation increased cytotoxic activity in NK-LIL and matched NK-TIL. (**e**) Proportion of DNAM-1+ NK cells and (**f**) expression of DNAM-1 receptor in NK cells from IL-15 stimulated LIL and TIL. The non-parametric Wilcoxon matched-pairs signed-rank test was used to compare data.

TIL-NK cell cytotoxic activity was significantly reduced compared with matched LIL NK cells (Figure 6a). After treatment with IL-15, significantly increased NK cell degranulation was observed in both NK-LIL and NK-TIL compartments (Figure 6b,c). However, the NK-TIL cytotoxic potential remained significantly lower than that of NK-LIL (Figure 6d), despite IL-15 stimulation having restored the DNAM-1 expression in NK-TIL (Figure 6e,f).

Interestingly, IFN-γ production was comparable in NK-LIL and NK-TIL both before and after IL-15 stimulation (Figure S10a,b), suggesting that the deficiency of DNAM-1 activating function was limited to cytotoxic activity. IL-15 stimulated NK cell cytotoxicity for classical K562 target cells and was comparable in NK-LIL and NK-TIL (Figure S11a,c). Similar findings were observed using primary HCC cells as targets (Figure S11b,d). Thus, collectively, these findings suggest that lower DNAM-1 mediated cytotoxicity was not due to a deficient NK-TIL activity.

## 4. Discussion

HCC is the most common primary liver cancer, accounting for about 90% of cases. HCC is the sixth most common cancer worldwide, and the fourth leading cause of cancer-related deaths worldwide [28]. HCC treatment options have considerably improved over the last few years, ranging from surgical resection, loco-regional approaches (thermal ablation and transarterial chemoembolization), to liver transplantation or drugs, such as sorafenib or lenvatinib, for an advanced disease, and immunotherapeutic treatment with anti-PD-L1 and anti-VEGF [29]. However, the overall HCC mortality rate remains disturbingly high. Despite the wealth of information on molecular biology, tumor growth rate, surveillance, diagnosis, and management, there is currently only a scarcity of seminal studies addressing the immunopathogenesis of HCC, which may have important implications in the design of immunotherapeutic strategies. Several studies support a key role for innate immunity, particularly for NK cells, in response to HCC [30]. The anti-tumor NK cell activities are modulated by interactions between NK cells activating, or by inhibitory receptors and their ligands expressed or released by target cells. In primary liver cancers, many molecules seem to be involved in the modulation of NK cell response, leading to alterations of receptor/ligand axes. In relation to this, we observed impaired NKp30- and NKG2D-mediated NK cell cytotoxicity in the tumor compartment [24,25,31]. The expression in HCC cells of ligands for several NK cell receptors often correlates with an outcome of liver disease. For instance, reduced expression of the major histocompatibility complex class I chain-related protein A and B (MICA/B), the ligand of the NKG2D activating receptor, is associated with a significantly shorter disease-free and overall survival in patients with HCC compared with patients with preserved MICA expression [32,33]. HCC cells can variably express the CD155 molecule [27,34], which is the common ligand for both activating DNAM-1, as well as the inhibitory CD96 and TIGIT receptors [14]. Moreover, in a recent interesting study [35], a new functional interaction between the KIR2DL5 and the immune receptor CD155 regulates natural killer cell cytolysis, inhibiting tumor cell killing.

Here, we found that tumors with higher expression of CD155 were moderately differentiated and patients with CD155-expressing HCC showed a trend toward longer overall survival compared with those with no CD155 expression, akin to previous findings [26,27]. In contrast, Sun et al. [36] found that higher concentration of CD155 were associated with worsening disease status, decreased overall and disease-free survival in HCC patients. This discrepancy may depend upon the prevalence of positive or negative signals mediated by DNAM-1 or CD96 receptors, respectively. Indeed, patients with higher proportions of tumor-infiltrating CD96+ NK cells and CD155-expressing HCC cells had a worse outcome.

In our study, the expression of DNAM-1 in NK-TIL was lower than that of NK-LIL. In agreement with previous findings in the NKp30/B7-H6 axis from our laboratory [24], we found that DNAM-1 downregulation depended in the engagement of DNAM-1 by CD155, there also being an association with CD155 expression in HCC cells. Decreased DNAM-1 expression was related to impaired DNAM-1-mediated NK-TIL cytotoxic activity. Stimulation with IL-15 was unable to restore full functionality, despite the recovery of DNAM-1 expression and the significantly increased degranulation detected in NK-TIL. In contrast, IFN-γ production by NK-TIL was comparable to that of NK-LIL, suggesting the existence of a selective deficiency in DNAM-1-mediated cytotoxic function. This may be ascribed to a difference in the prevalence of several distinct NK cell subsets with committed functions. In investigating anti-tumor NK cell responses in the C57BL/6 mouse model, Rocca and collaborators [37] found a hierarchy of responses that were dependent on different intrinsic reactivities of NK cell subsets, defined by the coexpression of several receptors. For instance, the coexpression of CD27, NKG2A, and DNAM-1 identified a subset with relative cytotoxic specialization, whereas reciprocally, CD11b and KLRG1 defined the best IFN-γ producers. Although in humans this has not yet been described, we can hypothesize that in HCC, a DNAM-1-expressing NK cell subset specialized in cytolytic function could be selectively affected.

Cancer cells can also release a soluble form of CD155, and levels of sCD155 are increased in the sera of several cancer patients [12], including HCC patients [13]. In agreement with the latter, we observed higher levels of sCD155 in sera from patients with HCC which correlated with parameters of advanced liver disease. Patients with lower sCD155 had poorly differentiated HCC, whereas others showed lower sCD155 levels in early-stage HCC [13]. This discrepancy could be explained by differences in the clinical characteristics of patients and differences in the classification of tumors used to stratify patients. Indeed, we applied the histological grading (G1, G2, G3) rather than the China liver cancer staging (CNLC, early-stage I-II and late-stage III) used by Jin and collaborators [13]. Increased sCD155 serum levels were associated with lower expression of DNAM-1 in circulating NK cells from HCC patients. This could be due to endocytosis of receptors induced by soluble ligands, as observed for soluble MIC and B7-H6 that mediate internalization of the corresponding activating receptors NKG2D and NKp30, respectively [38,39]. Moreover, sCD155 could inhibit DNAM-1 activity and, consequently, the NK-mediated anti-tumor response. Actually, sCD155 may significantly suppress NK cell degranulation in vivo and in vitro, basically functioning as a neutralizing molecule, rather than a signal-transducer for DNAM-1 [40]. sCD155 may therefore compete with membrane-bound CD155 by binding to DNAM-1 and may play a role as a ligand decoy for DNAM-1 [40].

## 5. Conclusions

In conclusion, our findings suggest an alteration in the functional properties of the DNAM-1/CD155 axis in HCC, since DNAM-1 could be modulated downwards by cell-free or cell-associated CD155, leading to the impairment of the cytotoxic activity of NK cells. This can be a potential tumor escape mechanism determining the suppression of NK cells’ antitumor immunity. The DNAM-1/CD155 axis may therefore represent an attractive, druggable target for new immunotherapeutic approaches in HCC.

## Supplementary Materials

The following supporting information can be downloaded at: https://www.mdpi.com/article/10.3390/cancers14164060/s1, Table S1: Clinical characteristics of patients; Figure S1. Gating strategy to identify DNAM-1+ cells, Figure S2. Patient discrimination for the sCD155 levels, Figure S3. mRNA level of CD155 isoforms in tumor and matched non-tumor tissue, Figure S4. DNAM-1 is downregulated upon exposure to Huh 7.5 cell line, Figure S5. Expression of CD155 in tumor cells, Figure S6. Downregulation of DNAM-1 after exposure to primary HCC cells, Figure S7. Downregulation of DNAM-1 in enriched NK cells, Figure S8. Tumor-siRNA-CD155 transfected cells modulated the DNAM-1 expression, Figure S9. Anti-DNAM-1 mAb boosts NK cell degranulation. Figure S10. IFN-γ production by NK cell of LIL and TIL compartment, Figure S11. NK cell degranulation toward tumor cells.

## Abbreviations

PVR	Poliovirus receptor
DNAM-1	DNAX-associated molecule-1
PBMCs	Peripheral blood mononuclear cells
HDs	Healthy donors
NK	Natural killer
HCC	Hepatocellular carcinoma
NT	Non-tumor
qPCR	Quantitative real-time polymerase chain reaction
LIL	Liver-infiltrating lymphocytes
TIL	Tumor-infiltrating lymphocytes
ELISA	Enzyme-linked immunosorbent assay
NKG2D	Natural killer group 2 member d
MICA/B	MHC class I chain-related molecules (MIC)A/B
rADCC	Reverse antibody-dependent cellular cytotoxicity
mAb	Monoclonal antibody
MELD	Model of end-stage liver disease
FIB-4	Fibrosis-4
IFN	Interferon
