# Supplementary material for: Defective DNAM-1 Dependent Cytotoxicity in Hepatocellular Carcinoma-Infiltrating NK Cells
Source: Cancers (Basel). 2022 Aug 22;14(16):4060. doi: 10.3390/cancers14164060 (PMC9406989; doi:10.3390/cancers14164060)
Supplement: Supplementary file 1 [file cancers-14-04060-s001.zip › cancers-1807423-supplementary.pdf]

cancers

MDPI

Defective DNAM-1 Dependent Cytotoxicity in Hepatocellular Carcinoma-Infiltrating NK Cells

Stefania Mantovani, Stefania Varchetta, Dalila Mele, Roberta Maiello, Matteo Donadon, Cristiana Soldani, Barbara Franceschini, Guido Torzilli, Giuseppe Tartaglia, Marcello Maestri, Gaetano Piccolo, Matteo Barabino, Enrico Opocher, Stefano Bernuzzi, Mario U Mondelli* and Barbara Oliviero

Table S1. Clinical characteristics of patients.

	HCC	%	no HCC	%
Number of subjects	142		19	
Male/Female	114/28	80/20	10/9	53/47
Median age (years) - range	70 (36-87)	-	68 (47-77)	-
ALT (mU/ml) median, range	46 (11-253)	-	65 (12-254)	-
AST (mU/ml), median, range	55 (13-326)	-	73 (15-187)	-
APRI, median, range	0.85 (0.21-7.97)	-	1.76 (0.16-3.21)	-
FIB-4, median, range	3.09 (0.77-21.15)	-	5.35 (1.35-12.41)	-
PTLs (10^3/ul)	155 (22.7-518)	-	131 (72-414)	-
BCLC score			na	
A	74	52.1	-	-
B	36	25.4	-	-
C	32	22.5	-	-
CTP score				
A	131	92.3	18	94.7
B	11	7.7	1	5.3
C	0	0	0	0
MELD score				
6–8	85	60	14	73.7
9-17	57	40	5	26.3
HCC size** (mm), median, range	36.5 (8-280)	-	na	-
Tumour Grading			na	
G1	25	17.6	-	-
G2	61	43	-	-
G3	37	26.1	-	-
G1-G2	5	3.5	-	-
G2-G3	2	1.4	-	-
G3-G4	1	0.7	-	-
Unknown	11	7.7	-	-
Etiology				
HCV	93	65.5	19	100
HBV	19	13.4	0	0
HBV + HCV	2	1.4	0	0
Non Virological	28	19.7	0	0

*na: not applicable.

**Maximum nodule size.

ALT, alanine aminotransferase; AST, aspartate aminotransferase; APRI, aspartate aminotransferase to platelet ratio index; FIB-4, fibrosis-4; PTLs, platelets; BCLC, Barcelona Clinic Liver Cancer; CTP, Child-Turcotte-Pugh; MELD, Model for End Stage Liver Disease; HBV, hepatitis B virus; HCV, hepatitis C virus.

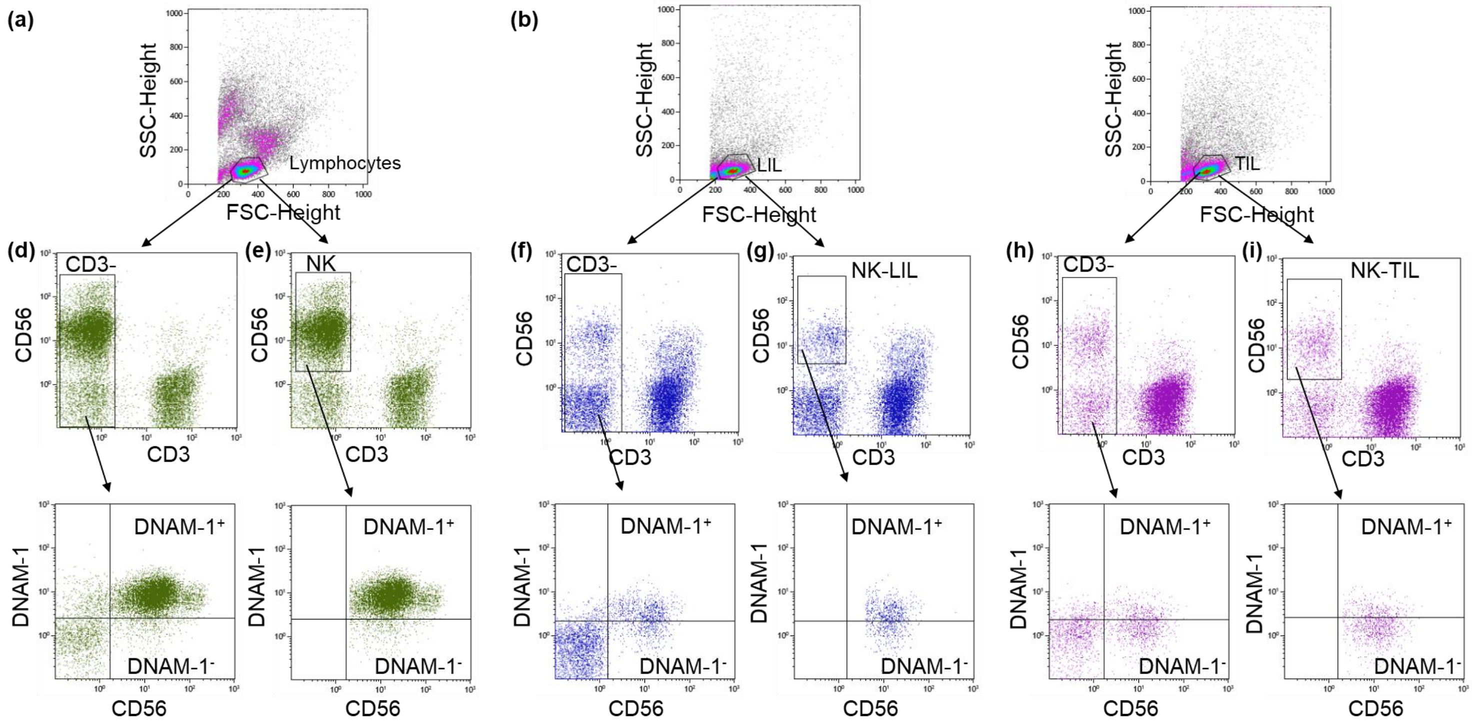

Figure S1. The gating strategy to identify DNAM-1+ cells. First, lymphocytes **(a)**, LIL **(b)** and TIL **(c)** were gated by FSC and SSC parameters. A gate on total CD3- **(d, f, h)** allows to discriminate between DNAM1^{+} and DNAM-1^{-} cells. Then, the same quadrants were used to identified DNAM-1+ cells among CD3-/CD56+ NK, NK-LIL and NK-TIL **(e, g, i)**.

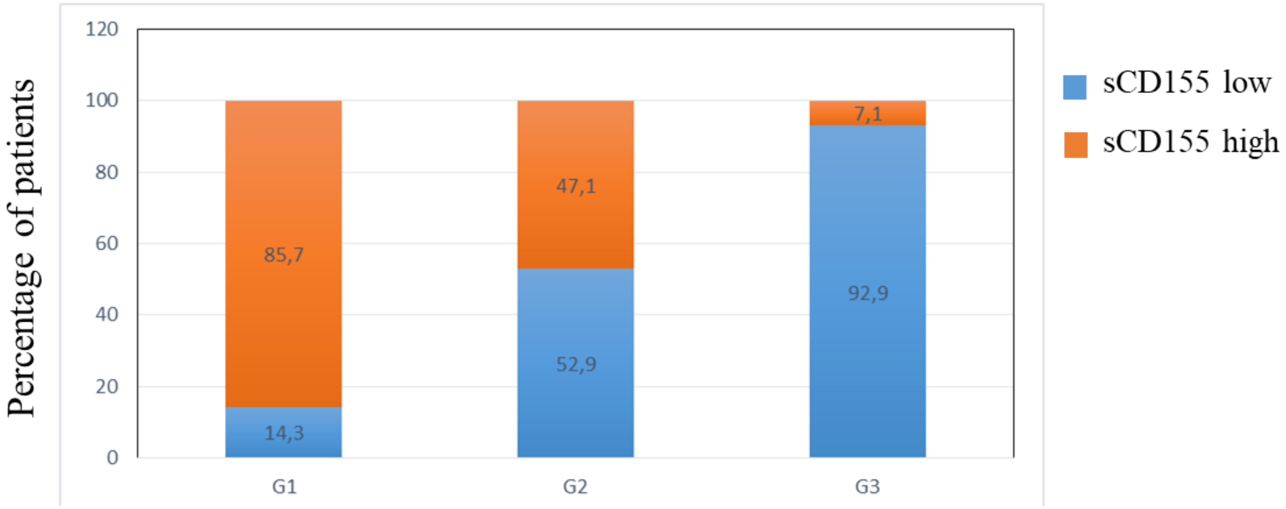

Figure S2. Patient discrimination for the sCD155 levels. The median value of sCD155 (26.83 ng/ml) was identified as cut-off to discriminate patients with high or low sCD155 levels. Patients with high or low sCD155 were then stratified according to the tumor differentiation grading (G1, G2, G3).

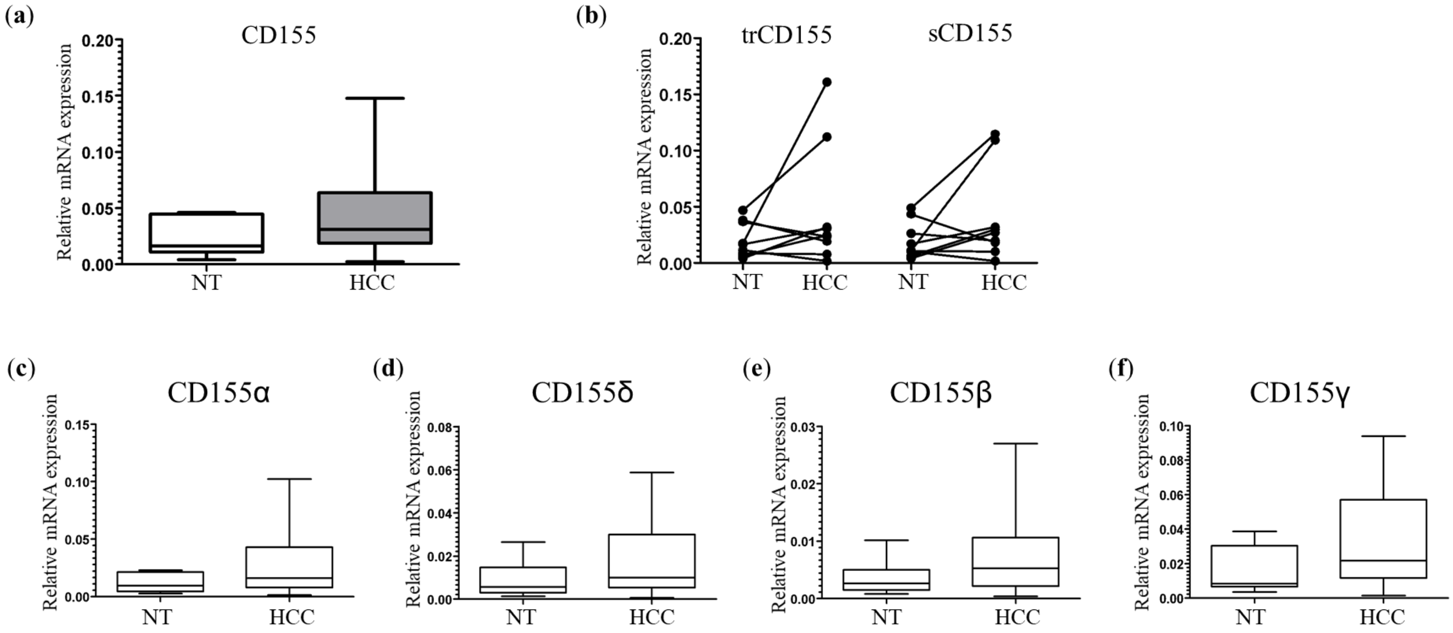

Figure S3. mRNA level of CD155 isoforms in tumor and matched non-tumor tissue. (a) Total CD155 mRNA was measured in non-tumor (NT) and matched tumor (HCC) tissue of 9 HCC patients. (**b**) CD155 mRNA level of trans-membrane α and δ (trCD155) or soluble β and γ (sCD155) isoforms. **(c)**, **(d)**, **(e)**, **(f)** mRNA level of single isoforms in matched NT and HCC tissues. Middle bars represent median values, box plots are 25% and 75% percentiles, whiskers are minimum and maximum values. The non parametric Wilcoxon matched pairs test was used to compare data.

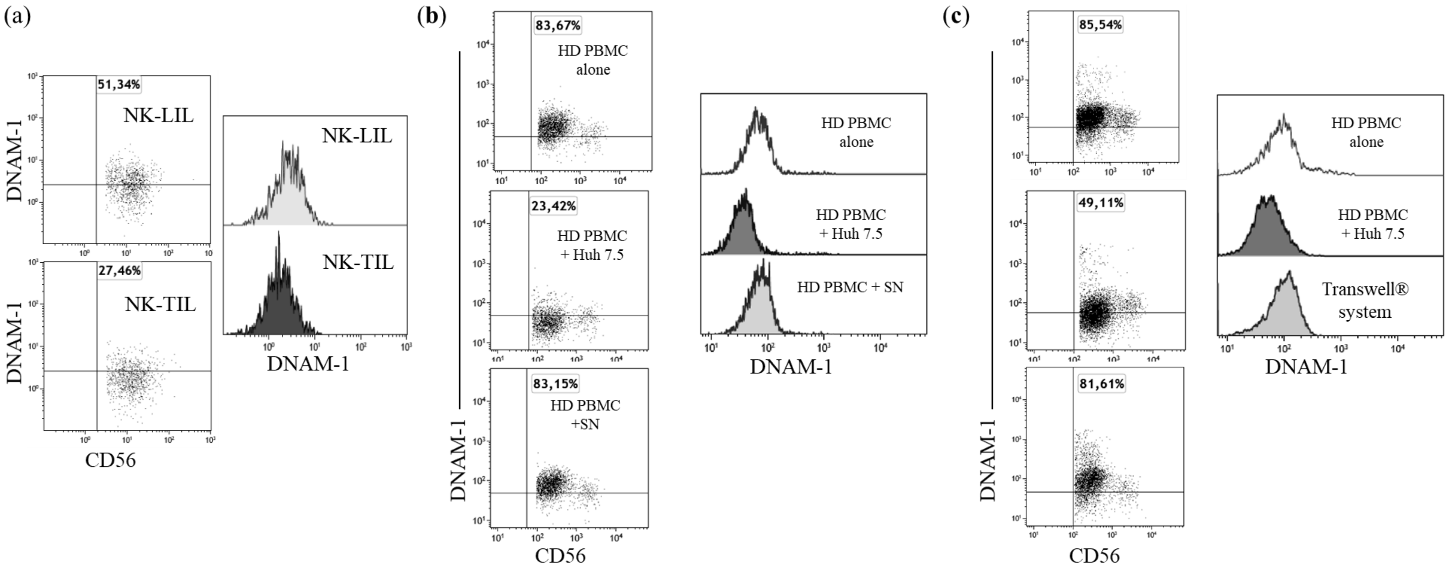

Figure S4. DNAM-1 is downregulated upon exposure to Huh 7.5 cell line. (a) DNAM-1 expression measured *ex vivo* by flow cytometry in NK of LIL and matched TIL compartment. **(b)** Dot plots and histograms showing the frequency of DNAM-1+ NK cells and DNAM-1 MFI of NK cells in the presence or absence of Huh 7.5 cells or cell-free supernatant (SN) from Huh 7.5 cell cultures. **(c)** Expression of DNAM-1 determined in PBMC alone and after exposure to Huh 7.5 cells or in the Transwell® system to prevent contact with Huh 7.5 cells.

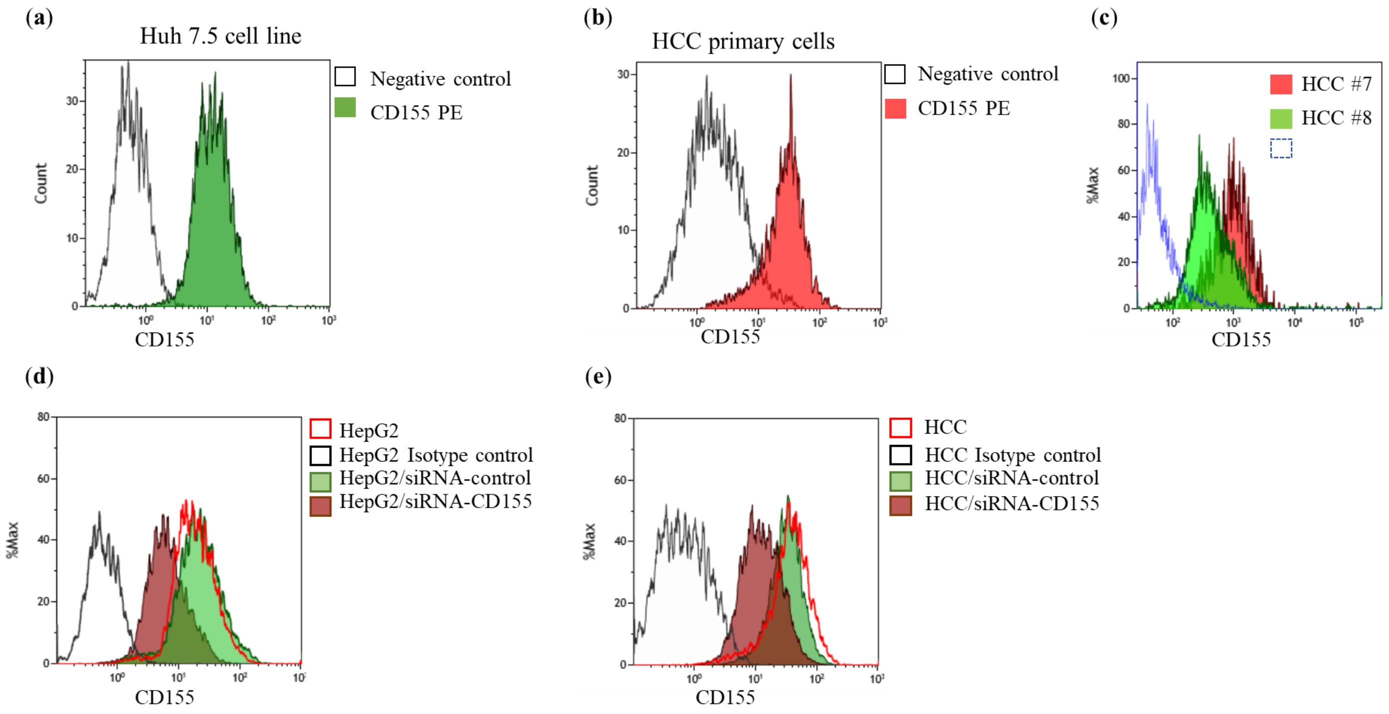

Figure S5. Expression of CD155 on tumor cells. CD155 expression was measured by flow cytometry on (**a**) Huh 7.5 cell line and (**b**) on a representative HCC primary cell line. (**c**) Different expression of CD155 on the HCC primary cells used in experiments with Transwell® system. siRNA-mediated knockdown of CD155 reduced CD155 expression on (**d**) HepG2-siRNA-tranfected cells and (**e**) on HCC-siRNA-transfected primary cells.

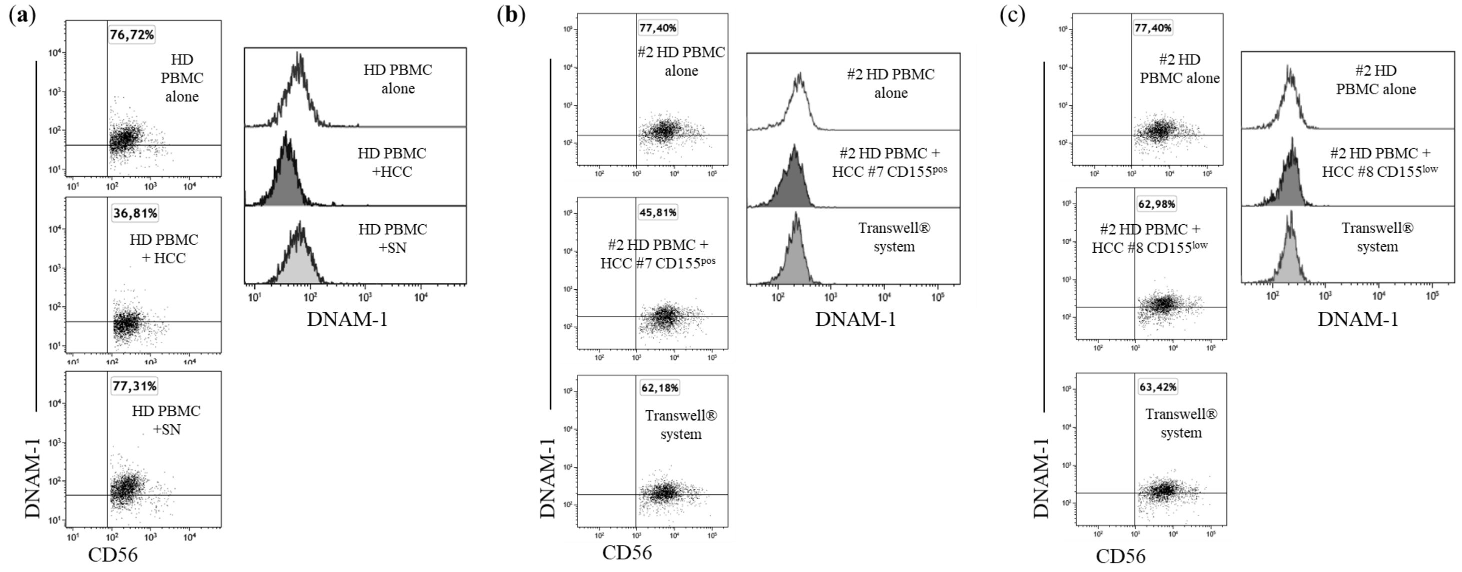

Figure S6. Downregulation of DNAM-1 after exposure to primary HCC cells. (a) Representative dot plots and histograms of DNAM-1 expression after co-culture of HD PBMC with primary HCC cells or with HCC cell culture SN. **(b)** DNAM-1 expression on NK cells from PBMC of a representative HD (#2) co-cultured with primary HCC cells expressing CD155 or in the Transwell® system. **(c)** The experiments were repeated using primary HCC cells expressing low levels CD155.

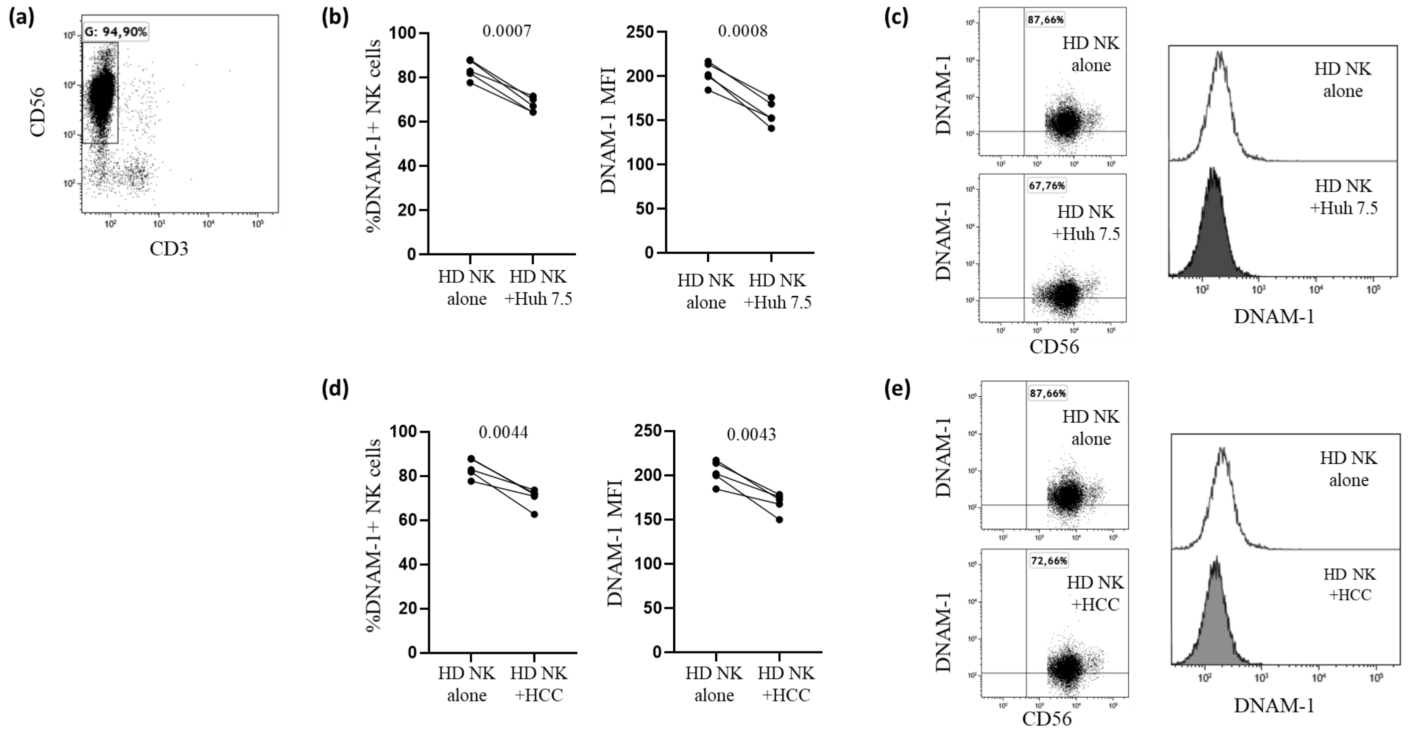

Figure S7. Downregulation of DNAM-1 on enriched NK cells. NK cells were enriched from PBMC of 5 HD subjects and co-cultured with Huh 7.5 or primary HCC cells. **(a)** Representative dot plot of NK cell enrichment NK. **(b)** Percentage of DNAM-1+ NK cells and **(d)** DNAM-1 expression without (HD NK alone) or in the presence of Huh 7.5 (HD NK + Huh 7.5) or HCC cells (HD NK + HCC). **(c)** and **(e)** Representative dot plots and histograms of DNAM-1 variations.

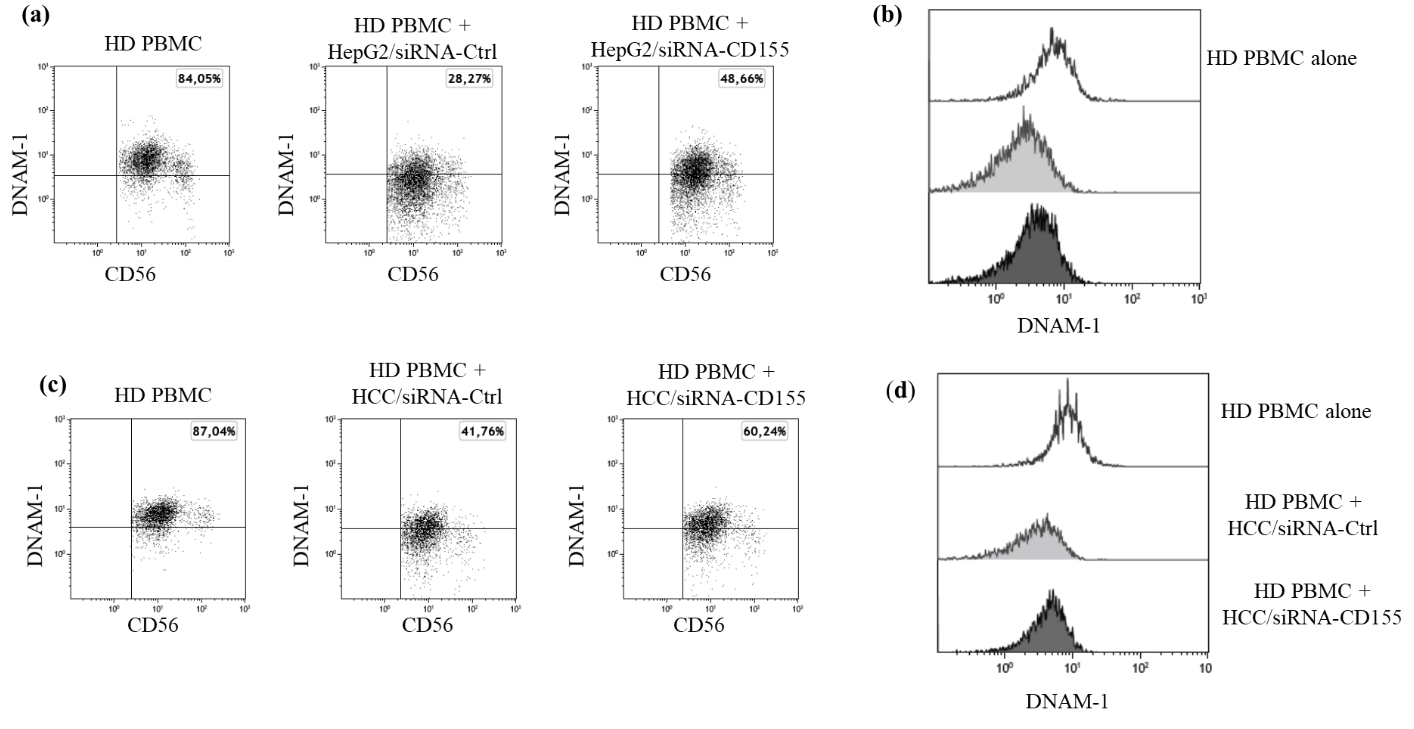

Figure S8. Tumor-siRNA-CD155 transfected cells modulated the DNAM-1 expression. (a) Representative dot plots of DNAM-1+ NK cells and **(b)** DNAM-1 MFI in HD PBMC exposed to siRNA-CD155- or siRNA-control-transfected

HepG2 cells. **(c, d)** PBMC from a representative HD were exposed to primary HCC cells transfected with siRNA-CD155 or siRNA-control.

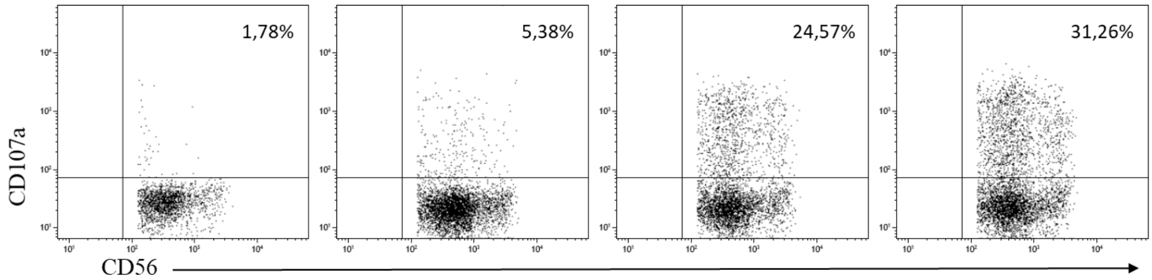

HD PBMC	+	+	+	+
FcγR+ P815 murine target cells	+	+	+	+
IL-15	-	+	+	+
Anti-DNAM-1 mAb	-	-	+ (0,1ug/ml)	+ (1ug/ml)

Figure S9. Anti-DNAM-1 mAb boosts NK cell degranulation. NK cell degranulation (%CD107a+ NK cells) was measured in unstimulated and IL-15 stimulated PMBC derived from one HD, in the presence of P815 murine target cells w/o anti-DNAM-1 mAb.

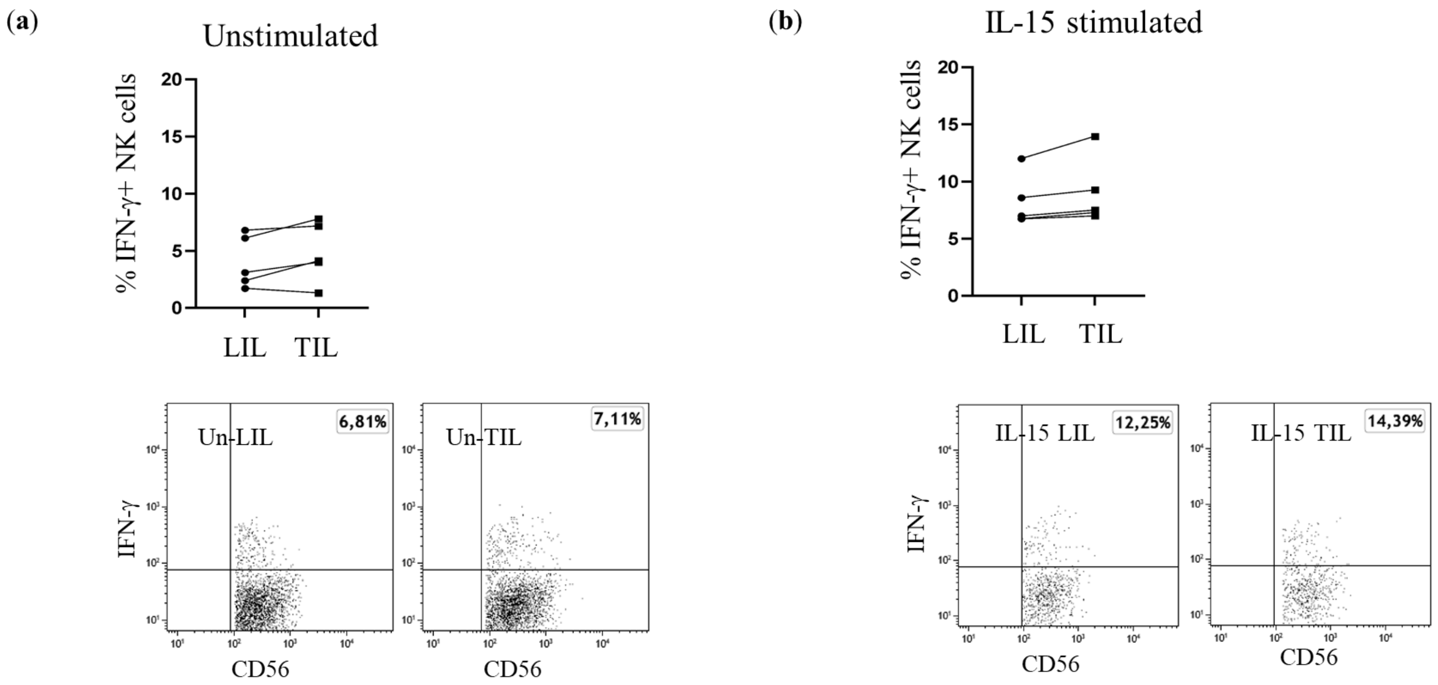

Figure S10. IFN-g production by NK cell of LIL and TIL compartment. IFN-g production by NK cells in unstimulated (**a**) or IL-15 stimulated (**b**) LIL and TIL from 5 HCC patients. Dot plots show representative IFN-g production from LIL and TIL before (Un-LIL, Un-TIL) and after (IL-15 LIL, IL-15 TIL) stimulation with IL-15.

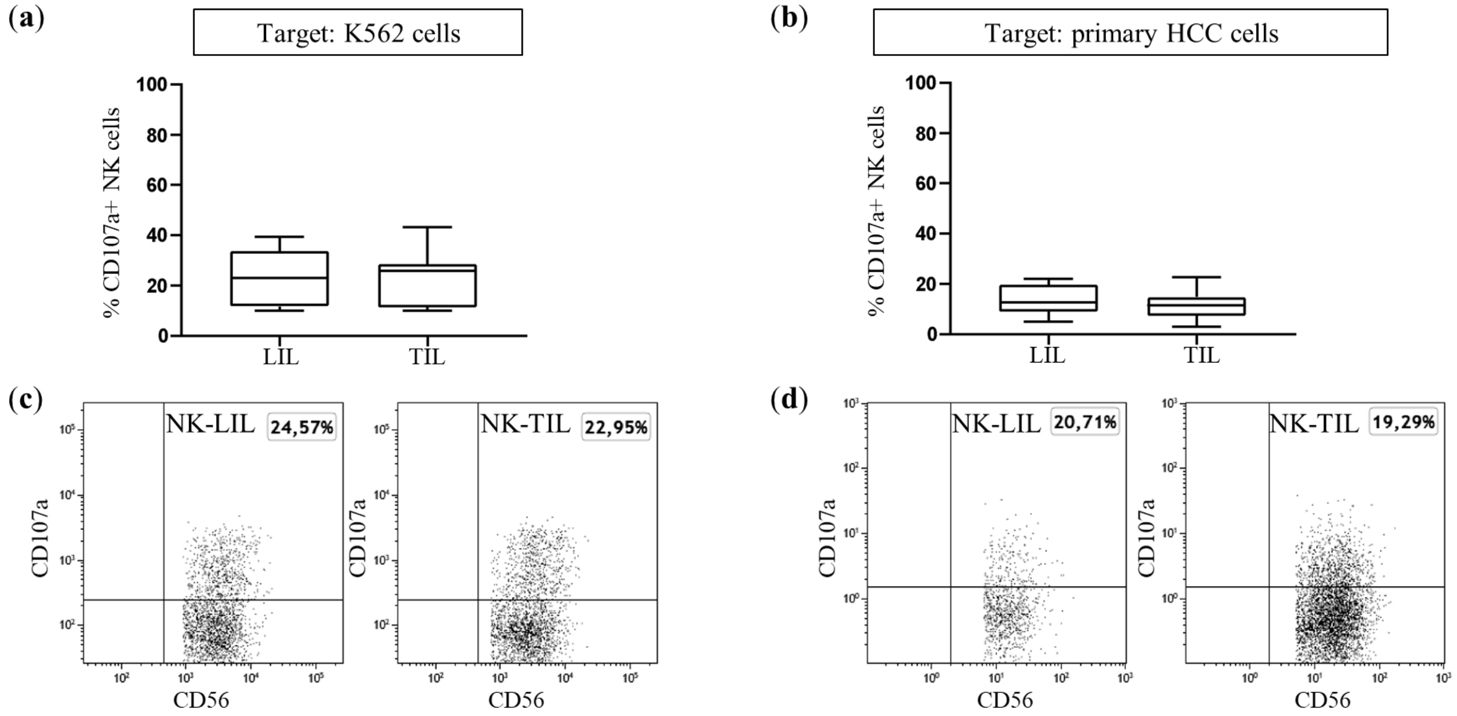

Figure S11. NK cell degranulation toward tumor cells. NK cell degranulation (%CD107a+ NK cells) was measured in IL-15 stimulated LIL and TIL toward the classical K562 cell line (**a**, n=10) or matched primary HCC cells (**b**, n=13). Middle bars represent median values, box plots are 25% and 75% percentiles, whiskers are minimum and maximum values. The non-parametric Wilcoxon matched-pairs signed rank test was used to compare data. (**c**), (**d**): Representative dot plots showing the proportions of CD107a+ NK cells in LIL and TIL.
